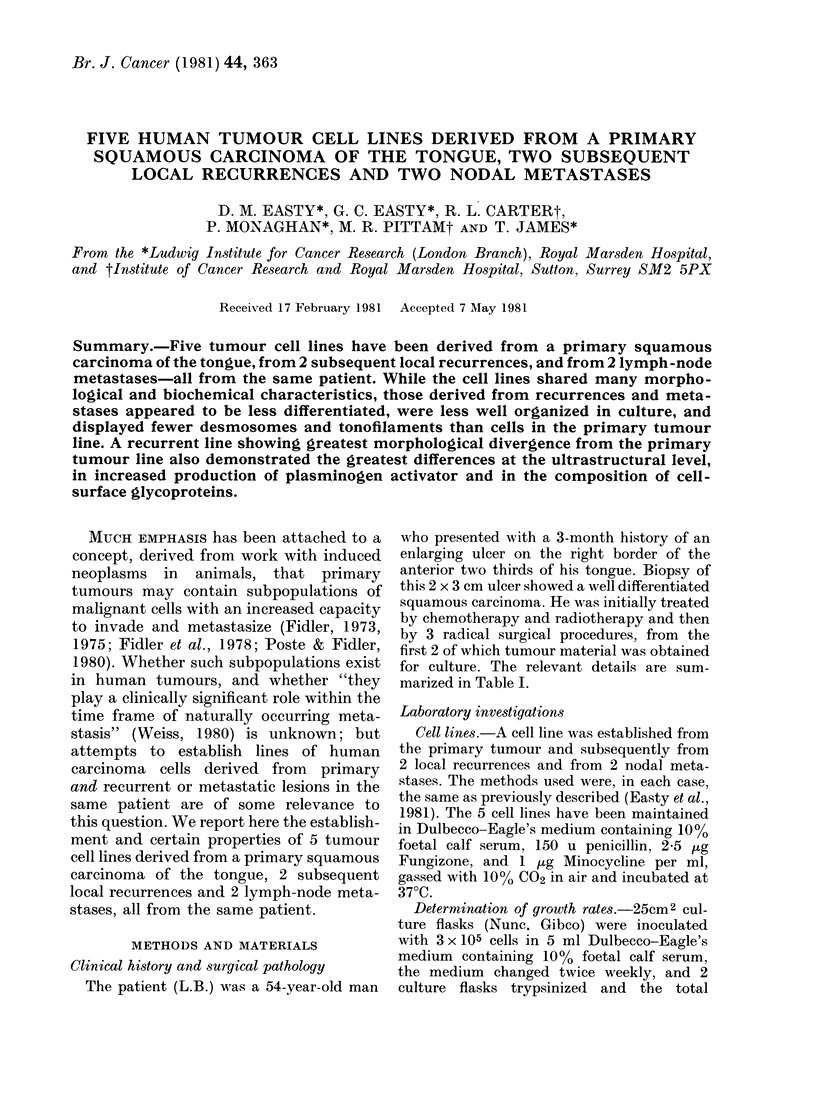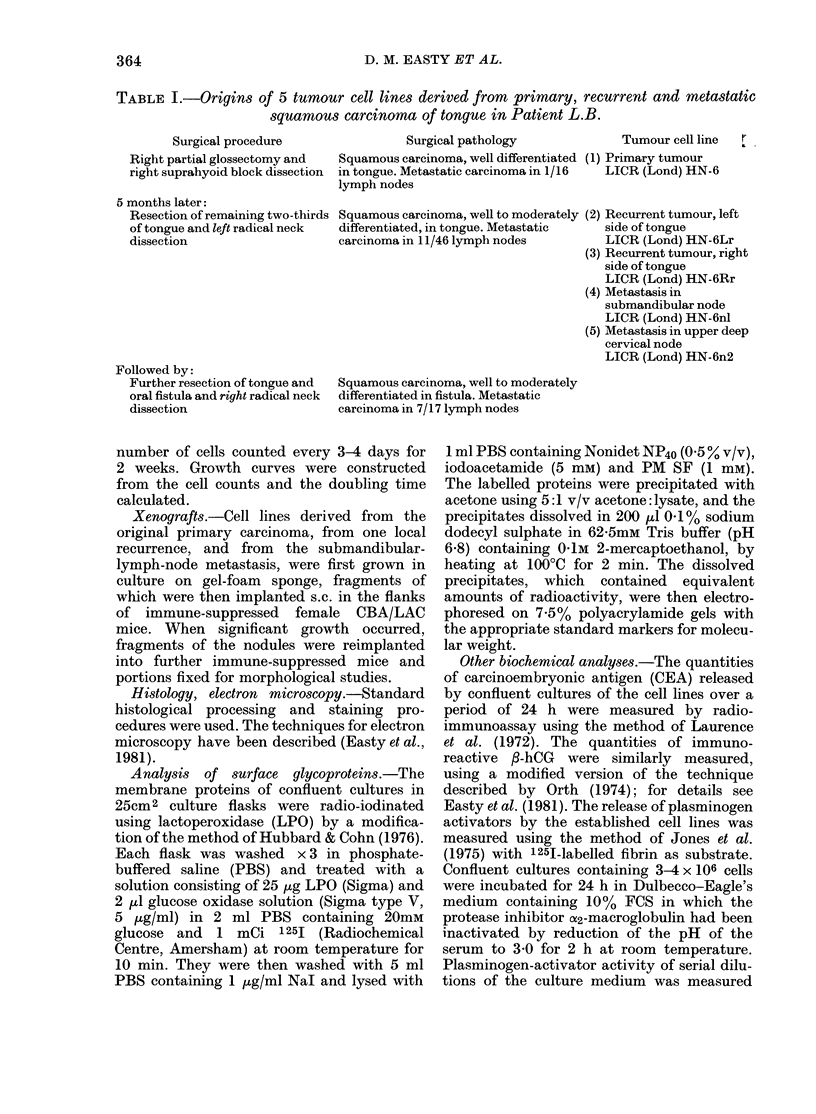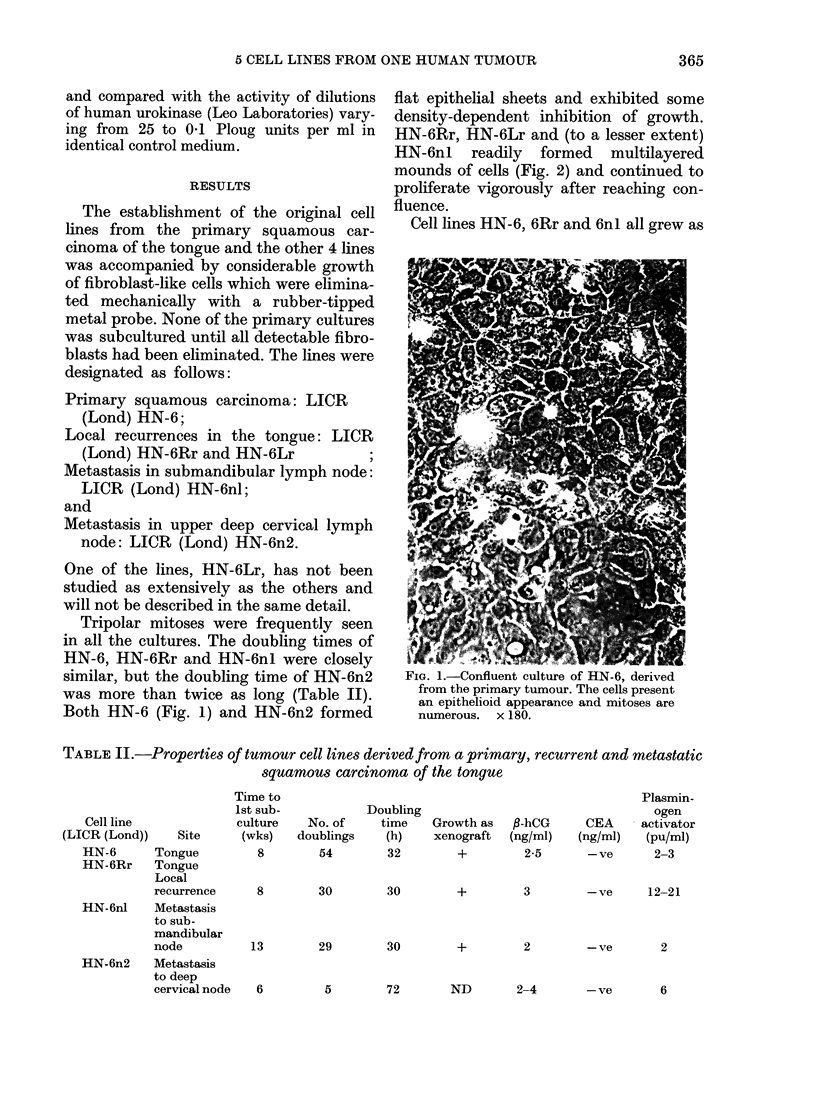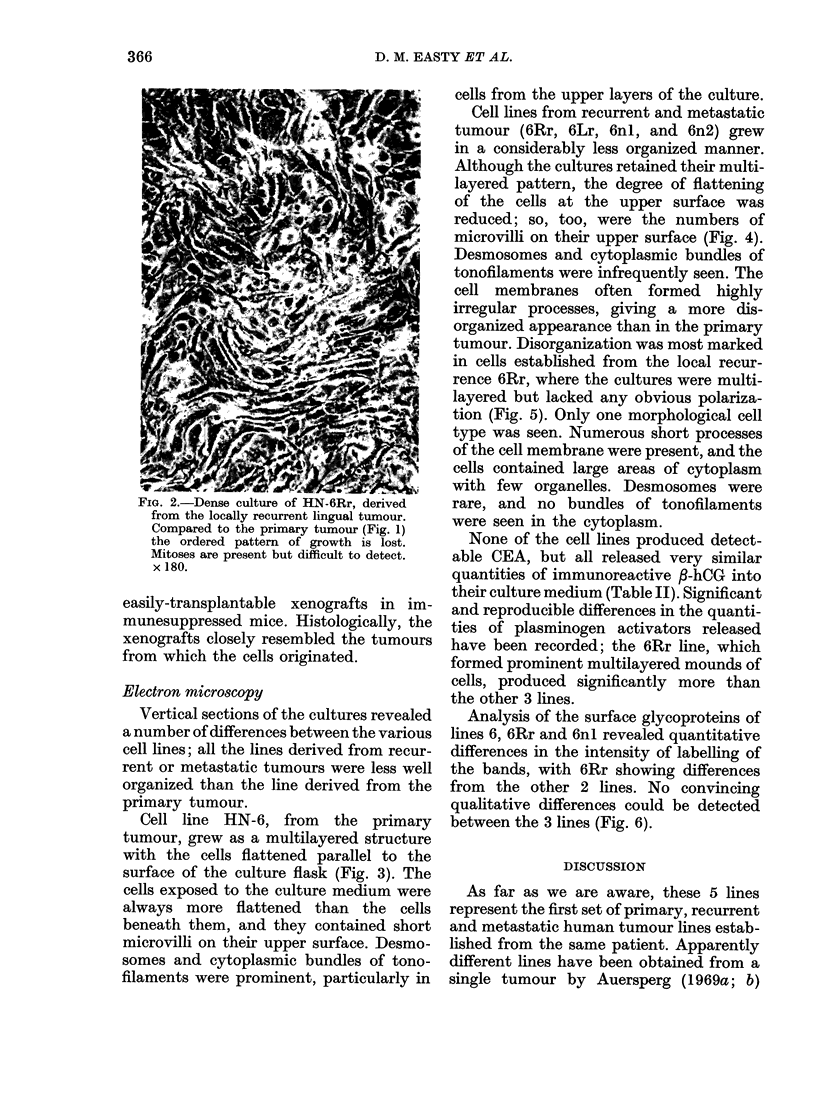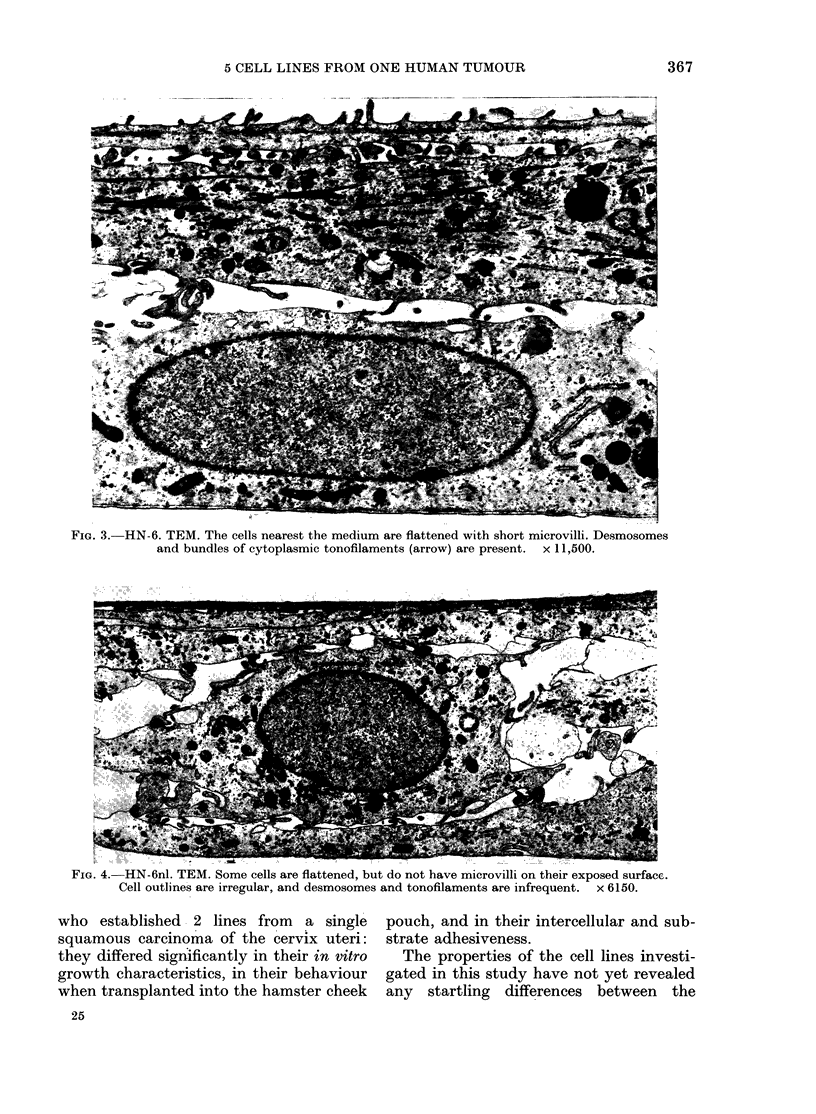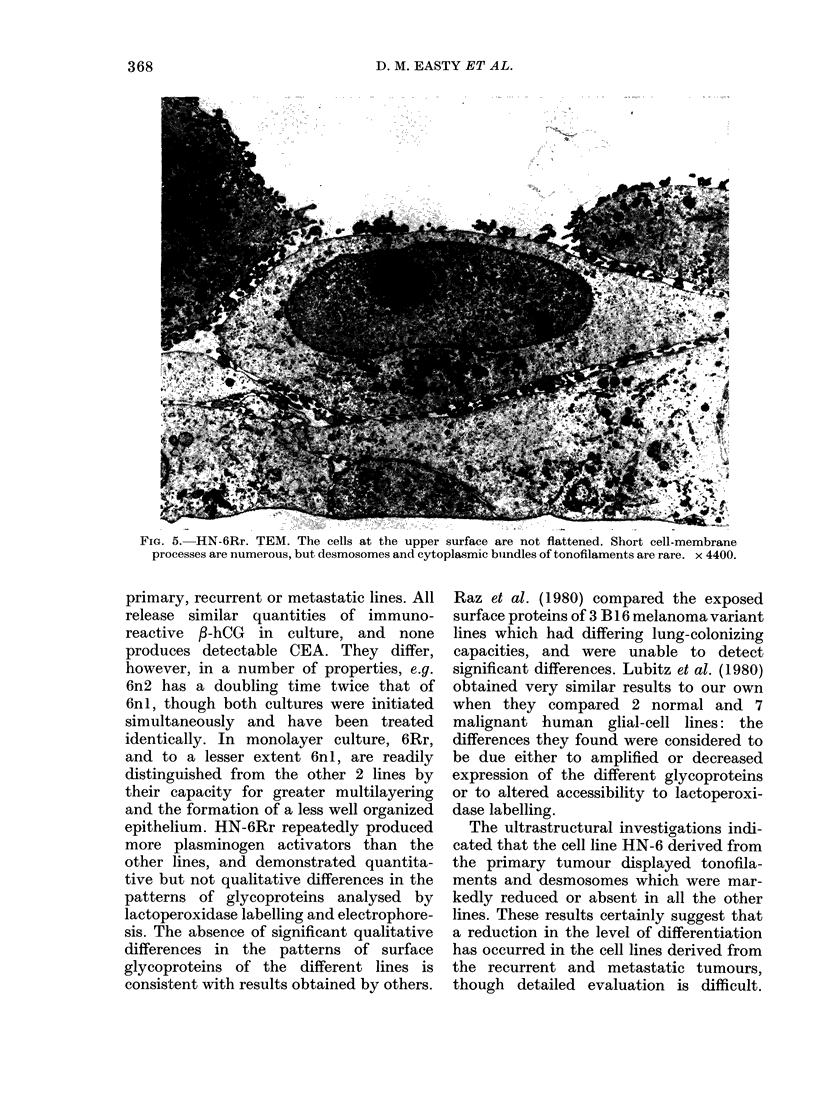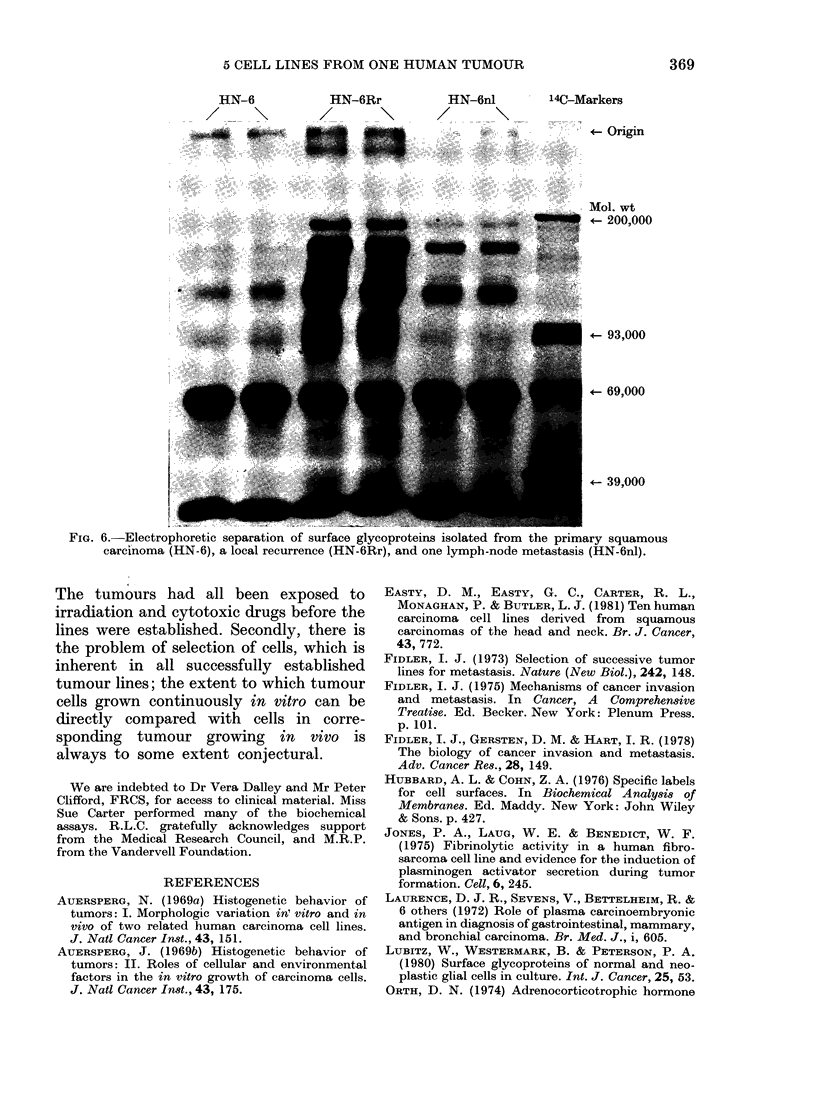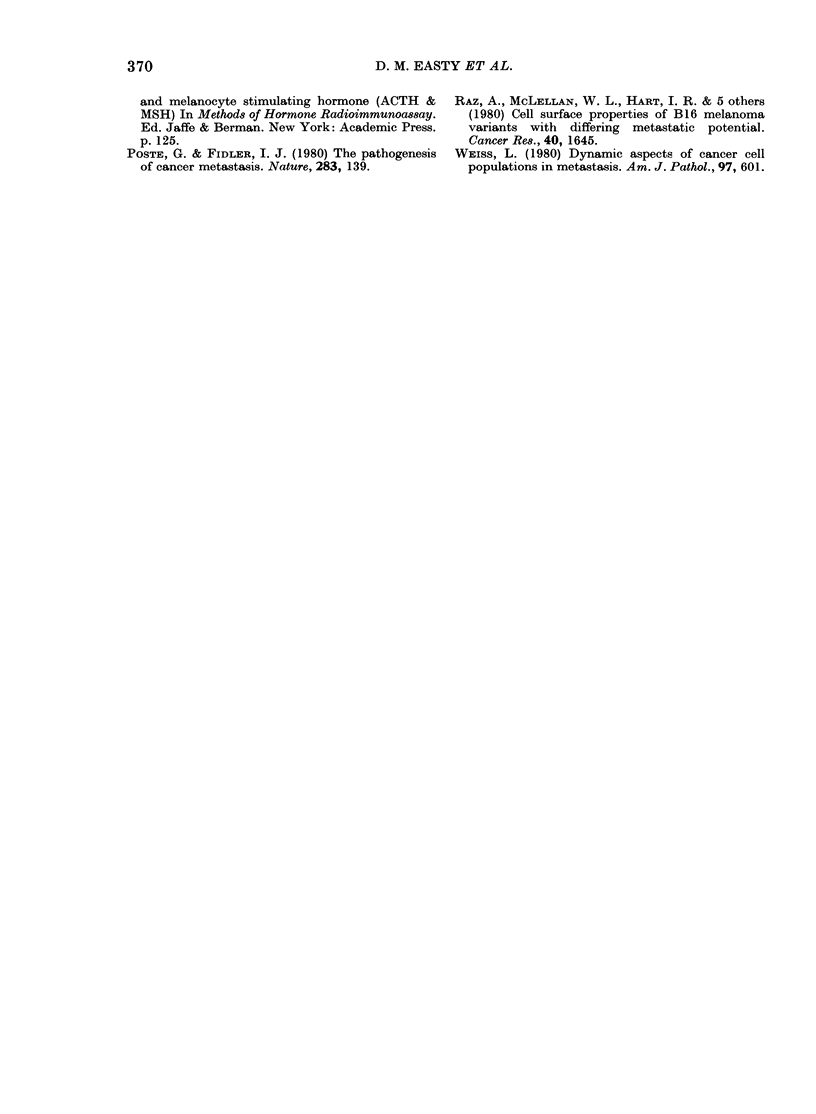# Five human tumour cell lines derived from a primary squamous carcinoma of the tongue, two subsequent local recurrences and two nodal metastases.

**DOI:** 10.1038/bjc.1981.193

**Published:** 1981-09

**Authors:** D. M. Easty, G. C. Easty, R. L. Carter, P. Monaghan, M. R. Pittam, T. James

## Abstract

**Images:**


*Br. J. Cancer* (1981) **44**, 363

### FIVE HUMAN TUMOUR CELL LINES DERIVED FROM A PRIMARY SQUAMOUS CARCINOMA OF THE TONGUE, TWO SUBSEQUENT LOCAL RECURRENCES AND TWO NODAL METASTASES

D. M. EASTY*, G. C. EASTY*, R. L. CARTER†, P. MONAGHAN*, M. R. PITTAM† AND T. JAMES*

*From the *Ludwig Institute for Cancer Research (London Branch), Royal Marsden Hospital, and †Institute of Cancer Research and Royal Marsden Hospital, Sutton, Surrey SM2 5PX*

Received 17 February 1981 Accepted 7 May 1981

**Summary.—Five tumour cell lines have been derived from a primary squamous carcinoma of the tongue, from 2 subsequent local recurrences, and from 2 lymph-node metastases—all from the same patient. While the cell lines shared many morphological and biochemical characteristics, those derived from recurrences and metastases appeared to be less differentiated, were less well organized in culture, and displayed fewer desmosomes and tonofilaments than cells in the primary tumour line. A recurrent line showing greatest morphological divergence from the primary tumour line also demonstrated the greatest differences at the ultrastructural level, in increased production of plasminogen activator and in the composition of cell-surface glycoproteins.**

MUCH EMPHASIS has been attached to a concept, derived from work with induced neoplasms in animals, that primary tumours may contain subpopulations of malignant cells with an increased capacity to invade and metastasize (Fidler, 1973, 1975; Fidler *et al.*, 1978; Poste & Fidler, 1980). Whether such subpopulations exist in human tumours, and whether "they play a clinically significant role within the time frame of naturally occurring metastasis" (Weiss, 1980) is unknown; but attempts to establish lines of human carcinoma cells derived from primary *and* recurrent or metastatic lesions in the same patient are of some relevance to this question. We report here the establishment and certain properties of 5 tumour cell lines derived from a primary squamous carcinoma of the tongue, 2 subsequent local recurrences and 2 lymph-node metastases, all from the same patient.

### METHODS AND MATERIALS

### *Clinical history and surgical pathology*

The patient (L.B.) was a 54-year-old man who presented with a 3-month history of an enlarging ulcer on the right border of the anterior two thirds of his tongue. Biopsy of this 2 × 3 cm ulcer showed a well differentiated squamous carcinoma. He was initially treated by chemotherapy and radiotherapy and then by 3 radical surgical procedures, from the first 2 of which tumour material was obtained for culture. The relevant details are summarized in Table I.

### *Laboratory investigations*

*Cell lines.*—A cell line was established from the primary tumour and subsequently from 2 local recurrences and from 2 nodal metastases. The methods used were, in each case, the same as previously described (Easty *et al.*, 1981). The 5 cell lines have been maintained in Dulbecco–Eagle's medium containing 10% foetal calf serum, 150 u penicillin, 2·5 μg Fungizone, and 1 μg Minocycline per ml, gassed with 10% $CO_2$ in air and incubated at 37°C.

*Determination of growth rates.*—25cm² culture flasks (Nunc, Gibco) were inoculated with $3 \times 10^5$ cells in 5 ml Dulbecco–Eagle's medium containing 10% foetal calf serum, the medium changed twice weekly, and 2 culture flasks trypsinized and the total

TABLE I.—*Origins of 5 tumour cell lines derived from primary, recurrent and metastatic squamous carcinoma of tongue in Patient L.B.*

| Surgical procedure | Surgical pathology | Tumour cell line |
|---|---|---|
| Right partial glossectomy and right suprahyoid block dissection | Squamous carcinoma, well differentiated in tongue. Metastatic carcinoma in 1/16 lymph nodes | (1) Primary tumour LICR (Lond) HN-6 |
| 5 months later: Resection of remaining two-thirds of tongue and *left* radical neck dissection | Squamous carcinoma, well to moderately differentiated, in tongue. Metastatic carcinoma in 11/46 lymph nodes | (2) Recurrent tumour, left side of tongue LICR (Lond) HN-6Lr |
| | | (3) Recurrent tumour, right side of tongue LICR (Lond) HN-6Rr |
| | | (4) Metastasis in submandibular node LICR (Lond) HN-6nl |
| | | (5) Metastasis in upper deep cervical node LICR (Lond) HN-6n2 |
| Followed by: Further resection of tongue and oral fistula and *right* radical neck dissection | Squamous carcinoma, well to moderately differentiated in fistula. Metastatic carcinoma in 7/17 lymph nodes | |

number of cells counted every 3–4 days for 2 weeks. Growth curves were constructed from the cell counts and the doubling time calculated.

*Xenografts.*—Cell lines derived from the original primary carcinoma, from one local recurrence, and from the submandibular-lymph-node metastasis, were first grown in culture on gel-foam sponge, fragments of which were then implanted s.c. in the flanks of immune-suppressed female CBA/LAC mice. When significant growth occurred, fragments of the nodules were reimplanted into further immune-suppressed mice and portions fixed for morphological studies.

*Histology, electron microscopy.*—Standard histological processing and staining procedures were used. The techniques for electron microscopy have been described (Easty *et al.*, 1981).

*Analysis of surface glycoproteins.*—The membrane proteins of confluent cultures in $25cm^2$ culture flasks were radio-iodinated using lactoperoxidase (LPO) by a modification of the method of Hubbard & Cohn (1976). Each flask was washed $\times 3$ in phosphate-buffered saline (PBS) and treated with a solution consisting of 25 $\mu$g LPO (Sigma) and 2 $\mu$l glucose oxidase solution (Sigma type V, 5 $\mu$g/ml) in 2 ml PBS containing 20mM glucose and 1 mCi $^{125}I$ (Radiochemical Centre, Amersham) at room temperature for 10 min. They were then washed with 5 ml PBS containing 1 $\mu$g/ml NaI and lysed with 1 ml PBS containing Nonidet $NP_{40}$ (0·5% v/v), iodoacetamide (5 mM) and PM SF (1 mM). The labelled proteins were precipitated with acetone using 5:1 v/v acetone: lysate, and the precipitates dissolved in 200 $\mu$l 0·1% sodium dodecyl sulphate in 62·5mM Tris buffer (pH 6·8) containing 0·1M 2-mercaptoethanol, by heating at 100°C for 2 min. The dissolved precipitates, which contained equivalent amounts of radioactivity, were then electrophoresed on 7·5% polyacrylamide gels with the appropriate standard markers for molecular weight.

*Other biochemical analyses.*—The quantities of carcinoembryonic antigen (CEA) released by confluent cultures of the cell lines over a period of 24 h were measured by radioimmunoassay using the method of Laurence *et al.* (1972). The quantities of immunoreactive $\beta$-hCG were similarly measured, using a modified version of the technique described by Orth (1974); for details see Easty *et al.* (1981). The release of plasminogen activators by the established cell lines was measured using the method of Jones *et al.* (1975) with $^{125}I$-labelled fibrin as substrate. Confluent cultures containing $3–4 \times 10^6$ cells were incubated for 24 h in Dulbecco–Eagle's medium containing 10% FCS in which the protease inhibitor $\alpha_2$-macroglobulin had been inactivated by reduction of the pH of the serum to 3·0 for 2 h at room temperature. Plasminogen-activator activity of serial dilutions of the culture medium was measured and compared with the activity of dilutions of human urokinase (Leo Laboratories) varying from 25 to 0·1 Ploug units per ml in identical control medium.

### RESULTS

The establishment of the original cell lines from the primary squamous carcinoma of the tongue and the other 4 lines was accompanied by considerable growth of fibroblast-like cells which were eliminated mechanically with a rubber-tipped metal probe. None of the primary cultures was subcultured until all detectable fibroblasts had been eliminated. The lines were designated as follows:

Primary squamous carcinoma: LICR (Lond) HN-6;
Local recurrences in the tongue: LICR (Lond) HN-6Rr and HN-6Lr ;
Metastasis in submandibular lymph node: LICR (Lond) HN-6nl;
and
Metastasis in upper deep cervical lymph node: LICR (Lond) HN-6n2.

One of the lines, HN-6Lr, has not been studied as extensively as the others and will not be described in the same detail.

Tripolar mitoses were frequently seen in all the cultures. The doubling times of HN-6, HN-6Rr and HN-6nl were closely similar, but the doubling time of HN-6n2 was more than twice as long (Table II). Both HN-6 (Fig. 1) and HN-6n2 formed flat epithelial sheets and exhibited some density-dependent inhibition of growth. HN-6Rr, HN-6Lr and (to a lesser extent) HN-6nl readily formed multilayered mounds of cells (Fig. 2) and continued to proliferate vigorously after reaching confluence.

Cell lines HN-6, 6Rr and 6nl all grew as

FIG. 1.—Confluent culture of HN-6, derived from the primary tumour. The cells present an epithelioid appearance and mitoses are numerous. ×180.

TABLE II.—*Properties of tumour cell lines derived from a primary, recurrent and metastatic squamous carcinoma of the tongue*

| Cell line (LICR (Lond)) | Site | Time to 1st subculture (wks) | No. of doublings | Doubling time (h) | Growth as xenograft | $\beta$-hCG (ng/ml) | CEA (ng/ml) | Plasminogen activator (pu/ml) |
|---|---|---|---|---|---|---|---|---|
| HN-6 | Tongue | 8 | 54 | 32 | + | 2·5 | −ve | 2–3 |
| HN-6Rr | Tongue Local recurrence | 8 | 30 | 30 | + | 3 | −ve | 12–21 |
| HN-6nl | Metastasis to submandibular node | 13 | 29 | 30 | + | 2 | −ve | 2 |
| HN-6n2 | Metastasis to deep cervical node | 6 | 5 | 72 | ND | 2–4 | −ve | 6 |

FIG. 2.—Dense culture of HN-6Rr, derived from the locally recurrent lingual tumour. Compared to the primary tumour (Fig. 1) the ordered pattern of growth is lost. Mitoses are present but difficult to detect. ×180.

easily-transplantable xenografts in immunesuppressed mice. Histologically, the xenografts closely resembled the tumours from which the cells originated.

*Electron microscopy*

Vertical sections of the cultures revealed a number of differences between the various cell lines; all the lines derived from recurrent or metastatic tumours were less well organized than the line derived from the primary tumour.

Cell line HN-6, from the primary tumour, grew as a multilayered structure with the cells flattened parallel to the surface of the culture flask (Fig. 3). The cells exposed to the culture medium were always more flattened than the cells beneath them, and they contained short microvilli on their upper surface. Desmosomes and cytoplasmic bundles of tonofilaments were prominent, particularly in cells from the upper layers of the culture.

Cell lines from recurrent and metastatic tumour (6Rr, 6Lr, 6n1, and 6n2) grew in a considerably less organized manner. Although the cultures retained their multilayered pattern, the degree of flattening of the cells at the upper surface was reduced; so, too, were the numbers of microvilli on their upper surface (Fig. 4). Desmosomes and cytoplasmic bundles of tonofilaments were infrequently seen. The cell membranes often formed highly irregular processes, giving a more disorganized appearance than in the primary tumour. Disorganization was most marked in cells established from the local recurrence 6Rr, where the cultures were multilayered but lacked any obvious polarization (Fig. 5). Only one morphological cell type was seen. Numerous short processes of the cell membrane were present, and the cells contained large areas of cytoplasm with few organelles. Desmosomes were rare, and no bundles of tonofilaments were seen in the cytoplasm.

None of the cell lines produced detectable CEA, but all released very similar quantities of immunoreactive $\beta$-hCG into their culture medium (Table II). Significant and reproducible differences in the quantities of plasminogen activators released have been recorded; the 6Rr line, which formed prominent multilayered mounds of cells, produced significantly more than the other 3 lines.

Analysis of the surface glycoproteins of lines 6, 6Rr and 6n1 revealed quantitative differences in the intensity of labelling of the bands, with 6Rr showing differences from the other 2 lines. No convincing qualitative differences could be detected between the 3 lines (Fig. 6).

### DISCUSSION

As far as we are aware, these 5 lines represent the first set of primary, recurrent and metastatic human tumour lines established from the same patient. Apparently different lines have been obtained from a single tumour by Auersperg (1969*a*; *b*)

FIG. 3.—HN-6. TEM. The cells nearest the medium are flattened with short microvilli. Desmosomes and bundles of cytoplasmic tonofilaments (arrow) are present. × 11,500.

FIG. 4.—HN-6nl. TEM. Some cells are flattened, but do not have microvilli on their exposed surface. Cell outlines are irregular, and desmosomes and tonofilaments are infrequent. × 6150.

who established 2 lines from a single squamous carcinoma of the cervix uteri: they differed significantly in their *in vitro* growth characteristics, in their behaviour when transplanted into the hamster cheek pouch, and in their intercellular and substrate adhesiveness.

The properties of the cell lines investigated in this study have not yet revealed any startling differences between the

FIG. 5.—HN-6Rr. TEM. The cells at the upper surface are not flattened. Short cell-membrane processes are numerous, but desmosomes and cytoplasmic bundles of tonofilaments are rare. × 4400.

primary, recurrent or metastatic lines. All release similar quantities of immunoreactive $\beta$-hCG in culture, and none produces detectable CEA. They differ, however, in a number of properties, *e.g.* 6n2 has a doubling time twice that of 6n1, though both cultures were initiated simultaneously and have been treated identically. In monolayer culture, 6Rr, and to a lesser extent 6n1, are readily distinguished from the other 2 lines by their capacity for greater multilayering and the formation of a less well organized epithelium. HN-6Rr repeatedly produced more plasminogen activators than the other lines, and demonstrated quantitative but not qualitative differences in the patterns of glycoproteins analysed by lactoperoxidase labelling and electrophoresis. The absence of significant qualitative differences in the patterns of surface glycoproteins of the different lines is consistent with results obtained by others. Raz *et al.* (1980) compared the exposed surface proteins of 3 B16 melanoma variant lines which had differing lung-colonizing capacities, and were unable to detect significant differences. Lubitz *et al.* (1980) obtained very similar results to our own when they compared 2 normal and 7 malignant human glial-cell lines: the differences they found were considered to be due either to amplified or decreased expression of the different glycoproteins or to altered accessibility to lactoperoxidase labelling.

The ultrastructural investigations indicated that the cell line HN-6 derived from the primary tumour displayed tonofilaments and desmosomes which were markedly reduced or absent in all the other lines. These results certainly suggest that a reduction in the level of differentiation has occurred in the cell lines derived from the recurrent and metastatic tumours, though detailed evaluation is difficult.

FIG. 6.—Electrophoretic separation of surface glycoproteins isolated from the primary squamous carcinoma (HN-6), a local recurrence (HN-6Rr), and one lymph-node metastasis (HN-6nl).

The tumours had all been exposed to irradiation and cytotoxic drugs before the lines were established. Secondly, there is the problem of selection of cells, which is inherent in all successfully established tumour lines; the extent to which tumour cells grown continuously *in vitro* can be directly compared with cells in corresponding tumour growing *in vivo* is always to some extent conjectural.

We are indebted to Dr Vera Dalley and Mr Peter Clifford, FRCS, for access to clinical material. Miss Sue Carter performed many of the biochemical assays. R.L.C. gratefully acknowledges support from the Medical Research Council, and M.R.P. from the Vandervell Foundation.

### REFERENCES

AUERSPERG, N. (1969*a*) Histogenetic behavior of tumors: I. Morphologic variation *in vitro* and *in vivo* of two related human carcinoma cell lines. *J. Natl Cancer Inst.*, **43**, 151.

AUERSPERG, J. (1969*b*) Histogenetic behavior of tumors: II. Roles of cellular and environmental factors in the *in vitro* growth of carcinoma cells. *J. Natl Cancer Inst.*, **43**, 175.

EASTY, D. M., EASTY, G. C., CARTER, R. L., MONAGHAN, P. & BUTLER, L. J. (1981) Ten human carcinoma cell lines derived from squamous carcinomas of the head and neck. *Br. J. Cancer*, **43**, 772.

FIDLER, I. J. (1973) Selection of successive tumor lines for metastasis. *Nature* (*New Biol.*), **242**, 148.

FIDLER, I. J. (1975) Mechanisms of cancer invasion and metastasis. In *Cancer, A Comprehensive Treatise*. Ed. Becker. New York: Plenum Press. p. 101.

FIDLER, I. J., GERSTEN, D. M. & HART, I. R. (1978) The biology of cancer invasion and metastasis. *Adv. Cancer Res.*, **28**, 149.

HUBBARD, A. L. & COHN, Z. A. (1976) Specific labels for cell surfaces. In *Biochemical Analysis of Membranes*. Ed. Maddy. New York: John Wiley & Sons. p. 427.

JONES, P. A., LAUG, W. E. & BENEDICT, W. F. (1975) Fibrinolytic activity in a human fibrosarcoma cell line and evidence for the induction of plasminogen activator secretion during tumor formation. *Cell*, **6**, 245.

LAURENCE, D. J. R., SEVENS, V., BETTELHEIM, R. & 6 others (1972) Role of plasma carcinoembryonic antigen in diagnosis of gastrointestinal, mammary, and bronchial carcinoma. *Br. Med. J.*, i, 605.

LUBITZ, W., WESTERMARK, B. & PETERSON, P. A. (1980) Surface glycoproteins of normal and neoplastic glial cells in culture. *Int. J. Cancer*, **25**, 53.

ORTH, D. N. (1974) Adrenocorticotrophic hormone and melanocyte stimulating hormone (ACTH & MSH) In *Methods of Hormone Radioimmunoassay*. Ed. Jaffe & Berman. New York: Academic Press. p. 125.

POSTE, G. & FIDLER, I. J. (1980) The pathogenesis of cancer metastasis. *Nature*, **283**, 139.

RAZ, A., MCLELLAN, W. L., HART, I. R. & 5 others (1980) Cell surface properties of B16 melanoma variants with differing metastatic potential. *Cancer Res.*, **40**, 1645.

WEISS, L. (1980) Dynamic aspects of cancer cell populations in metastasis. *Am. J. Pathol.*, **97**, 601.